# Comparison of conventional and rapid-acting antidepressants in a rodent probabilistic reversal learning task

**DOI:** 10.1177/2398212820907177

**Published:** 2020-02-23

**Authors:** Matthew P. Wilkinson, John P. Grogan, Jack R. Mellor, Emma S. J. Robinson

**Affiliations:** 1 School of Physiology, Pharmacology and Neuroscience, University of Bristol, Bristol, UK; 2 Nuffield Department of Clinical Neurosciences, University of Oxford, Oxford, UK

**Keywords:** Antidepressants, major depressive disorder, probabilistic reversal learning, ketamine, cognitive flexibility, Q-learning, feedback sensitivity, citalopram

## Abstract

Deficits in reward processing are a central feature of major depressive disorder with patients exhibiting decreased reward learning and altered feedback sensitivity in probabilistic reversal learning tasks. Methods to quantify probabilistic learning in both rodents and humans have been developed, providing translational paradigms for depression research. We have utilised a probabilistic reversal learning task to investigate potential differences between conventional and rapid-acting antidepressants on reward learning and feedback sensitivity. We trained 12 rats in a touchscreen probabilistic reversal learning task before investigating the effect of acute administration of citalopram, venlafaxine, reboxetine, ketamine or scopolamine. Data were also analysed using a Q-learning reinforcement learning model to understand the effects of antidepressant treatment on underlying reward processing parameters. Citalopram administration decreased trials taken to learn the first rule and increased win-stay probability. Reboxetine decreased win-stay behaviour while also decreasing the number of rule changes animals performed in a session. Venlafaxine had no effect. Ketamine and scopolamine both decreased win-stay probability, number of rule changes performed and motivation in the task. Insights from the reinforcement learning model suggested that reboxetine led animals to choose a less optimal strategy, while ketamine decreased the model-free learning rate. These results suggest that reward learning and feedback sensitivity are not differentially modulated by conventional and rapid-acting antidepressant treatment in the probabilistic reversal learning task.

## Introduction

Reward learning (RL), the ability of reward to modulate future behaviour, is believed to contribute to the aetiology and treatment of depression (Der-Avakian et al., 2015; Vrieze et al., 2013). Probabilistic reward learning (PLT) and probabilistic reversal learning tasks (PRLT) have been used to study RL and the behavioural response to positive and negative feedback in humans and animals (Bari et al., 2010; Murphy et al., 2003; Slaney et al., 2018). Patients with major depressive disorder (MDD) show increased sensitivity to misleading feedback but respond normally to accurate negative feedback (Murphy et al., 2003; Taylor Tavares et al., 2008). MDD patients and patients in remission also have an impaired ability to integrate reward information over time (Pechtel et al., 2013; Pizzagalli et al., 2008).

Translation of human PLTs into rodent paradigms (Bari et al., 2010; Der-Avakian et al., 2013) provides an opportunity to probe the link between RL and depressive behaviour. Two types of tasks are commonly used: PLTs and PRLTs, the reversal learning version also providing a measure of cognitive flexibility. Utilising a PRLT, Bari et al. (2010) observed that chronic 5-mg/kg citalopram treatment increased positive feedback sensitivity (PFS) while acutely and bidirectionally modulating reversal performance and negative feedback sensitivity (NFS). Drozd et al. (2018) further characterised the PRLT with a range of conventional antidepressants and observed no effects with escitalopram and venlafaxine treatment, although mirtazapine decreased RL performance. The rapid-acting antidepressant, ketamine, has also been investigated in the PRLT with Rychlik et al. (2017) reporting decreased sensitivity to misleading negative feedback following treatment. Another PLT, the Response Bias Probabilistic Reward Task, has also been developed for rodents, and it has been shown to be sensitive to dopaminergic manipulations whereby amphetamine enhanced but pramipexole impaired RL (Der-Avakian et al., 2013). Antidepressants have not yet been assessed in this task.

Modulation of reward-related behaviour has been relatively widely studied in both animal models and humans (Lewis et al., 2019; Robinson and Roiser, 2016; Slaney et al., 2018). In traditional rodent models of anhedonia, chronic stress-induced impairments in reward sensitivity are reversed by chronic but not acute antidepressant treatments (Willner, 2017), while ketamine rapidly reverses these deficits (Yang et al., 2015). Recently, human emotional processing tasks have been translated into methods suitable for non-human species to study reward-related cognitive biases in rodent models (Hales et al., 2014; Robinson and Roiser, 2016). In the affective bias test (ABT), an assay probing how affective biases modulate learning and memory, conventional antidepressant treatment induces a positive bias during learning of new substrate-reward associations but does not ameliorate previously learnt negative biases (Stuart et al., 2013, 2015). Conversely, ketamine treatment was found to block negative biases but have no effect upon new learning. The judgement bias task (JBT) investigates how cognitive biases alter the valuation of ambiguous information. Within the JBT, ketamine rapidly increases optimistic responses towards the ambiguous cue, while conventional antidepressant treatment requires 2 weeks of treatment for an effect (Hales et al., 2017). Taken together, these findings suggest that different underlying neuropsychological process contribute to reward-related behaviours, and these are differentially modulated in models of depression and in response to delayed versus rapid-acting antidepressants.

In this study, we sought to compare the effects of conventional monoaminergic antidepressants and rapid-acting antidepressants upon behaviour in the PRLT. We tested the conventional antidepressants citalopram, venlafaxine and reboxetine alongside the rapid-acting antidepressants ketamine and scopolamine at doses previously used in the ABT and JBT. Drugs were administered acutely as our primary interest was the effects of the rapid-onset antidepressants upon RL. Some conventional antidepressants have been reported to acutely exacerbate anxiety (Urban et al., 2016); however, they still have acute antidepressant effects (Harmer et al., 2009; Stuart et al., 2013), and acute dosing allows comparison with previous PRLT studies. We additionally analysed data using a Q-learning model to probe parameter changes underlying differences in RL performance. By dissociating the neuropsychological mechanisms underlying differential responses to conventional and rapid-acting antidepressant treatment, this will aid in the development of future antidepressant compounds with both long-lasting efficacy and a rapid-onset of action.

## Methods

### Animals and housing

Twelve male Lister-hooded rats (Harlan, UK) were housed in pairs within enriched laboratory cages (55 × 35 × 21 cm) containing sawdust, paper bedding, red Perspex houses (30 × 17 × 10 cm), cotton rope and cardboard tubes. Sample size was estimated based on previous studies using a similar task and manipulations (Bari et al., 2010). Animals weighed on average 272 g at the start of training and 419 g prior to the commencement of drug study experiments. Rats were kept in temperature-controlled conditions (21 ± 1°C) and under a 12:12 h reverse light–dark cycle (lights off at 08:00 h). Water was available ad libitum in the home cage, but animals were mildly food restricted to no less than 90% of their free-feeding weight matched to a normal growth curve (≈18 g of food per rat/day laboratory chow (LabDiet, PMI Nutrition International)). All dosing and behavioural testing was carried out in the animals’ active phase between 09:00 and 18:00 h. All experiments were carried out in accordance with local institutional guidelines (University of Bristol Animal Welfare and Ethical Review Board), the UK Animals (Scientific procedures) Act of 1986 and the European Parliament and Council Directive of 22 September 2010 (2010/63/EU).

### Apparatus

Behavioural testing was carried out in touchscreen operant boxes (Med Associates, USA) containing a magazine delivering 45-mg reward pellets (Test Diet, Sandown Scientific, UK), house light, tone generator and infrared touchscreen panel with three windows within which animals could respond. The system was controlled by KLimbic Software (Conclusive Solutions Ltd, UK), and output files were then decoded in a custom MATLAB (MathWorks Inc version R2017a, USA) programme (see ‘Data analysis and output measures’).

### Behavioural task

**Figure 1.**
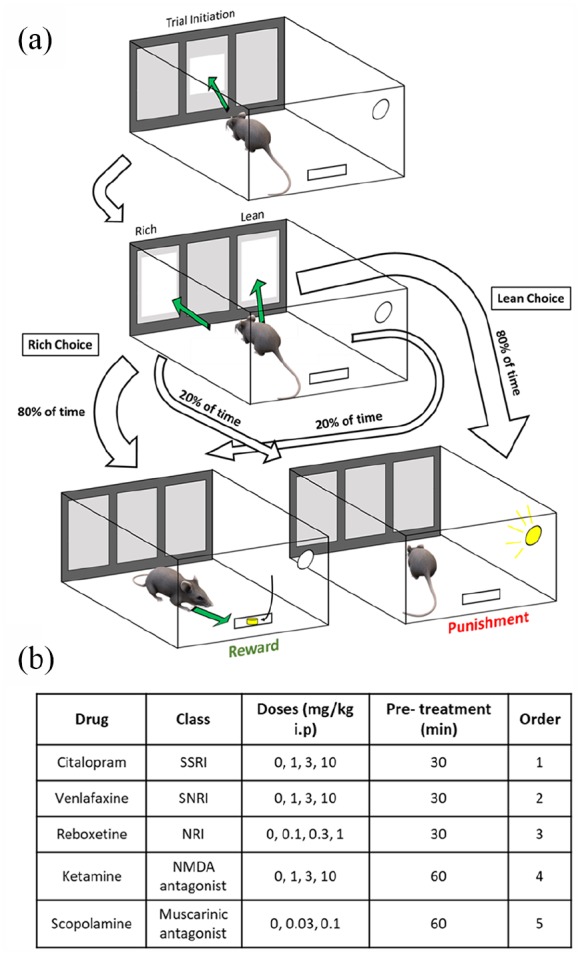
Overview of experimental protocol. (a) Schematic of all possible routes for a single successful trial in the PRLT task. Probabilities of each outcome are depicted by the width of each arrow. Green arrows represent an animal making an action, while white arrows depict transfer from one stage of a trial to the next. If no response was detected within 10 s of an animal pressing the initiation square, then this was classed as an omission and animals received a 5-s timeout. (b) Details of pharmacological studies, order refers to the sequence in which individual drug studies were carried out.

The PRLT was adapted for use on a touchscreen operant system from the original design by Bari et al. (2010) (see Figure 1(a) for a summary of the task design).

#### Training

Training was conducted in three stages. In stage 1, animals learnt to touch an initiation square presented in the centre window of the screen to receive a single reward pellet for a maximum of 120 trials or 30 min (whichever was reached first). In common with other touchscreen operant tasks, animals typically responded to the screen with their noses (Horner et al., 2013). Once animals reached criterion (completion of all 120 trials within a session for two consecutive sessions, mean time to train: 6.7 ± 0.51 sessions), they progressed onto stage 2. In this phase, animals had to first press the initiation square before then pressing either of two stimuli simultaneously presented in the left and right window to receive reward (max 200 trials or 40 min). Animals were deemed to reach criteria when they completed 80% of trials for two consecutive sessions (mean time to train: 2.25 ± 0.13 sessions). Rats then progressed onto the main spatial probabilistic reversal learning protocol.

#### Behavioural testing

In the PRLT, animals had to first press an initiation stimulus in the centre of the screen before then choosing to respond to either a left or right spatial stimulus. There was no time limit for the response to the initiation screen enabling animals to self-pace the task. Stimuli were probabilistically rewarded such that the ‘rich’ stimulus had an 80% chance of reward and the ‘lean’ stimulus had only a 20% chance of reward. Once an animal had selected a stimulus, they were either presented with a reward pellet in the magazine (once animals had retrieved the reward, the initiation screen illuminated and they could begin the next trial) or punished with no reward and a timeout of 5 s with the house light on. If animals did not make a choice of stimuli within 10 s after trial initiation, this was classified as an omission and animals received a timeout of 5 s during which time the house light was illuminated. Following eight consecutive ‘rich’ stimulus choices, the contingencies switched so that the spatial location previously associated with the ‘rich’ stimulus was now associated with the ‘lean’ stimulus and vice versa. Animals were permitted to serially reverse throughout a session (max 200 trials or 40 min). The spatial location of the ‘rich’ stimulus at the start of a session was consistent across sessions and counterbalanced across animals. Changing the location of the rich stimulus at the start of a session did not have any effect of overall task performance (see Supplemental Figure S2). Training was deemed to have been complete when animals’ performance had stabilised in the main output parameters of interest, allowing drug study experiments to commence (no main effect of session over last five sessions of training in rule changes, win-stay probability, lose-shift probability and initiation reaction time, see Supplemental Figure S1).

### Experimental design

Acute dose–response studies were conducted in a blinded, within-subject fully counterbalanced design with all animals receiving every dose of drug. Treatment groups were allocated through use of a fully randomised design containing four treatment groups (except for the scopolamine study where three groups were used) with each group having the treatments in a different order. The conventional antidepressants citalopram hydrobromide (Selective serotonin reuptake inhibitor (SSRI), 1, 3, 10 mg/kg; t = −30 m; HelloBio, UK), venlafaxine hydrochloride (serotonin–noradrenaline reuptake inhibitor (SNRI), 1, 3, 10 mg/kg; t = −30 m; HelloBio, UK) and reboxetine mesylate hydrate (noradrenaline reuptake inhibition (NRI), 0.1, 0.3, 1 mg/kg; t = −30 m; Sigma-Aldrich, UK) alongside the rapid-acting antidepressants ketamine hydrochloride (N-methyl-D-aspartate (NMDA) antagonist, 1, 3, 10 mg/kg; t = −60 m; Sigma-Aldrich, UK) and scopolamine hydrobromide (muscarinic antagonist, 0.03, 0.1 mg/kg; t = −60 m; Tocris, UK) were all dissolved in 0.9% saline and administered before the start of testing by intraperitoneal injection using a low-stress technique (Stuart and Robinson, 2015). Drug doses and pre-treatment times were chosen as to be clinically relevant and were based upon previous behavioural studies (Bari et al., 2010; Hales et al., 2017; Jones and Higgins, 1995; Stuart et al., 2013). All studies were carried out such that a baseline session always preceded a session when drug was administered, there was at least 2 days between each drug session and all animals completed at least five baseline sessions (minimum one week) between the end of a drug study and the commencement of the next to minimise any carryover effects of treatment. All drug studies were carried out in food-restricted animals, but a test using pre-feeding was not observed to have any effects on the main outcomes measured in the PRLT except from decreasing overall motivation (see Supplemental Figure S3).

### Data analysis and output measures

#### Parameters of interest

Output parameters were calculated to be consistent with previous PRLT studies (Bari et al., 2010; Rychlik et al., 2017). The number of rule changes and the first rule change trial were defined as the number of times an animal was able to successfully switch reward contingencies in a session and the trial at which an animal first achieved criterion for a rule change within a session, respectively. Win-stay behaviour was analysed as a proxy of PFS (how likely animals were to change their behaviour as a function of positive feedback), alongside lose-shift behaviour (how likely animals were to shift responding following negative feedback) which was examined as a proxy of NFS. Win-stay behaviour was defined as the probability upon receiving a reward at a stimulus that the rat would stay at that stimulus for the next trial as opposed to shifting to the opposite stimulus. Conversely, lose-shift behaviour was defined as the probability that following punishment at a stimulus, the rat would switch to the opposite stimulus for the next trial. Win-stay and lose-shift behaviour were additionally subdivided into either true or misleading feedback based upon whether the feedback given matched with the underlying rule of the task at the time. For example, if a rat was punished for selecting the ‘rich’ stimulus, this would be classed as misleading feedback but if it was rewarded for selecting the ‘rich’ stimulus, this would match with the underlying rule of the task and, therefore, be true feedback. Initiation reaction time was defined as the time taken for rats to respond to the presentation of the initiation square in the central window of the screen and was taken as a proxy for motivation to complete a trial.

#### Statistical analysis

Data were decoded and output measures calculated from KLimbic output files using a custom MATLAB (MathWorks Inc version R2017a, USA) programme before statistical analysis was conducted using SPSS (IBM version 24, USA) and output graphics constructed using GraphPad Prism 7 (GraphPad Software, USA). Sample size was calculated from previous experiments utilising similar behavioural tasks. Outlier exclusion was conducted blind to treatment, and animals that completed less than 50 trials were only analysed for the variables: number of rule changes and first rule change trial. Individual drug studies were analysed independently. Each behavioural parameter was analysed using one or two factor repeated measures analysis of variance (RM-ANOVA) with the factors treatment (containing each dose as a level) or treatment and feedback type, respectively (for true/misleading feedback analysis). All data were assessed for violations of sphericity using Mauchly’s test and where this was the case, degrees of freedom were adjusted using the Huynh–Feldt correction. Post hoc analysis was conducted using Sidak’s correction. Where data were non-normally distributed (assessed using Kolmogorov–Smirnov and Shapiro–Wilk tests), output variables were evaluated using the Friedman test with post hoc analysis carried out using Bonferroni-corrected Wilcoxon signed rank tests. Dotted lines indicate separate drug studies. A bracket and star(s) over multiple bars indicates a main effect of treatment, while star(s) over a single bar indicates a post hoc significant difference compared to vehicle treatment for that drug study. All data are shown as mean ± standard error of mean (SEM), *⩽0.05, **<0.01, ***<0.001, ****<0.0001.

### Modelling

Computational modelling of behavioural responses provides another method for evaluating the mechanism of antidepressant drug action by probing the underlying computational processes occurring in the brain to produce behaviour. The Q-learning model is one of the most widely used models for analysis of reinforcement learning and works using the same input information available to the animals to iterate through each trial and make decisions with the aim of maximising total reward. This computer ‘optimal strategy’ can then be compared with animal behaviour to allow estimation of both absolute RL performance and estimations of underlying RL parameters such as learning rate and softmax temperature. Theoretical accuracy was described as how well animals’ performance matched up to the model-predicted perfect strategy, allowing an estimation of absolute RL efficiency within a session. This is a different measure to observed accuracy which was a direct measure of task performance. β (the inverse temperature of the softmax equation) is related to how deterministic stimulus choices are, with high β values meaning that choices are made towards stimuli with higher estimated values, while low β values essentially mean that choices are random (Grogan et al., 2017). Data from both training and each drug study were analysed using a Q-learning model (Watkins and Dayan, 1992) adapted from Grogan et al. (2017) and discussed in detail there. Briefly, for each trial, the value (Q) of choosing each stimulus is updated with a proportion of the reward prediction error (RPE) – the difference between the reward expected from an action and the reward received. The proportion of the RPE used for updating is controlled by the learning rate (α) parameter. For acute drug studies, data for each individual rat were fit to two models: one containing a single learning rate (Qlearn1) and another containing a dual learning rate for positive and negative information (Qlearn2). The choice of model fit for each animal was made using Bayesian information criteria (BIC; Schwarz, 1978) when fitted to the vehicle data, with the model with the lowest BIC chosen. For every drug study, the single learning rate model (Qlearn1) was the better fitting model (Supplemental Figure S4). Once the model had been fit, these starting parameters were used to individually fit each dose and animal separately to create the output parameters.

## Results

### Effects of conventional antidepressants

**Figure 2.**
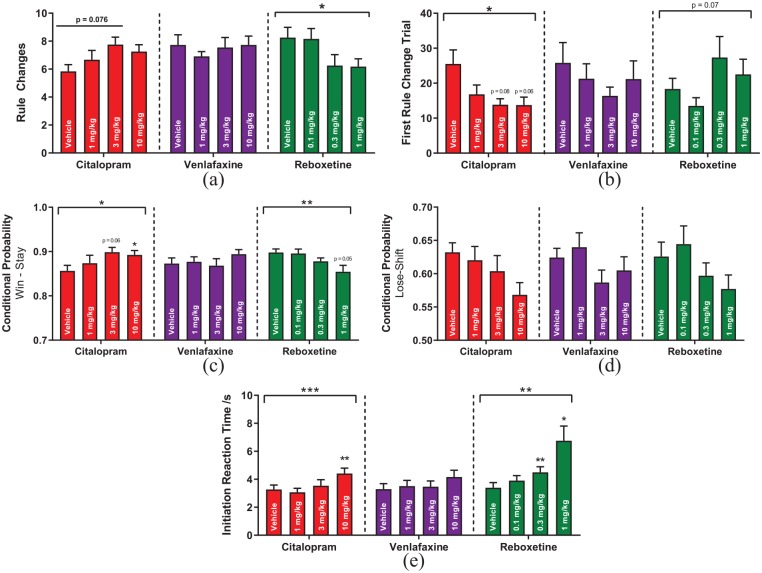
Effects of delayed antidepressant administration in the PRLT. (a) Rule changes completed within a session. (b) Trial at which animals first met the criterion for a rule change. (c) Win-stay probability. (d) Lose-shift probability. (e) Initiation reaction time. Dotted lines indicate separate drug studies.

Reboxetine decreased the number of rule changes (Figure 2(a)) within a session (RM-ANOVA, F_3,30_ = 3.31, p = 0.033), an overall integrative measure of RL performance. Although there was no main effect of citalopram treatment upon rule changes, there was a tendency for higher rule change performance when the first two doses of citalopram plus vehicle were analysed in isolation (RM-ANOVA, F_2,22_ = 2.91, p = 0.076). Citalopram reduced the trials taken to reach the first rule change (Friedman test, χ^2^(3) = 8.50, p = 0.037, Figure 2(b)), while there was also a trend towards reboxetine increasing the trials taken to reach the first rule change (Friedman test, χ^2^(3) = 7.02, p = 0.071). PFS, as measured by the proportion of win-stay behaviour (Figure 2(c)), was increased by citalopram treatment (RM-ANOVA, F_3,33_ = 3.81, p = 0.019). Surprisingly for an antidepressant, reboxetine decreased win-stay behaviour (RM-ANOVA, F_3,24_ = 6.16, p = 0.003). None of the conventional antidepressants tested had any effects upon lose-shift behaviour (Figure 2(d)), a measure of NFS. Both citalopram (RM-ANOVA, F_3,33_ = 8.02, p = 0.0004) and reboxetine (RM-ANOVA, F_1.25, 12.48_ = 11.17, p = 0.004) reduced motivation in the PRLT, as measured by the time taken to initiate a trial in the PRLT (Figure 2(e)). Venlafaxine (one animal excluded from study due to illness) had no effects on any variable measured in the PRLT. Reboxetine but not citalopram or venlafaxine also reduced the number of trials completed in a session (Friedman test, χ^2^(3) = 18.0, p = 0.0004, Supplemental Figure S5)

### Effects of rapid-acting antidepressants

**Figure 3.**
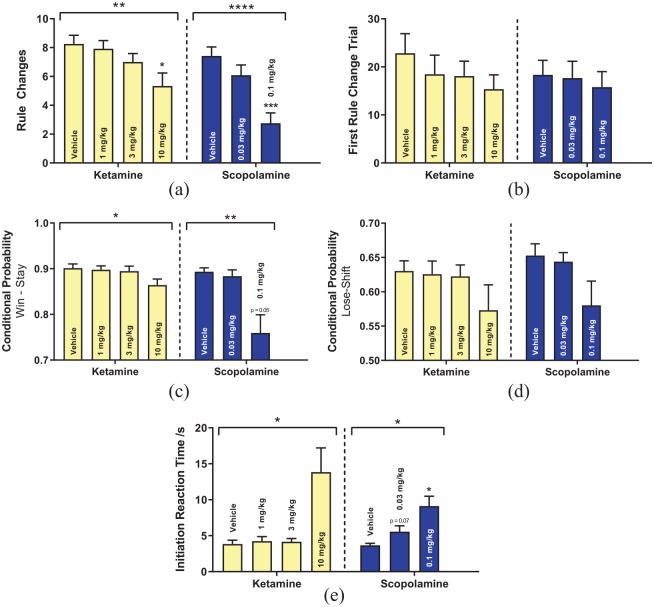
Effects of rapid-onset antidepressant administration in the PRLT. (a) Rule changes completed within a session. (b) Trial at which animals first met the criterion for a rule change. (c) Win-stay probability. (d) Lose-shift probability. (e) Initiation reaction time. Dotted lines indicate separate drug studies.

Both ketamine (RM-ANOVA, F_3,33_ = 5.697, p = 0.003) and scopolamine (RM-ANOVA, F_2,22_ = 16.23, p < 0.0001) reduced RL performance as measured by the number of rule changes completed in a session (Figure 3(a)). Neither drugs had any effect on the trial at which the first rule change was achieved (Figure 3(b)); however, both ketamine (RM-ANOVA, F_1.97, 19.66_ = 3.928, p = 0.037) and scopolamine (RM-ANOVA, F_2,14_ = 8.36, p = 0.004) decreased win-stay behaviour (Figure 3(c)). As seen with the conventional antidepressants, neither rapid-acting antidepressant tested had any effect on lose-shift behaviour (Figure 3(d)). Consistent with citalopram and reboxetine, both ketamine (RM-ANOVA, F_1.08, 9.68_ = 7.36, p = 0.021) and scopolamine (Friedman test, χ^2^(2) = 7.75, p = 0.021) also decreased motivation as measured by the time taken to initiate a trial (Figure 3(e)). Both ketamine (Friedman test, χ^2^(3) = 19.08, p = 0.0003, Supplemental Figure S5) and scopolamine (Friedman test, χ^2^(2) = 14.6, p = 0.0007, Supplemental Figure S5) additionally reduced trials completed by animals within a session.

### Effects of antidepressant treatment on true or misleading feedback

**Figure 4.**
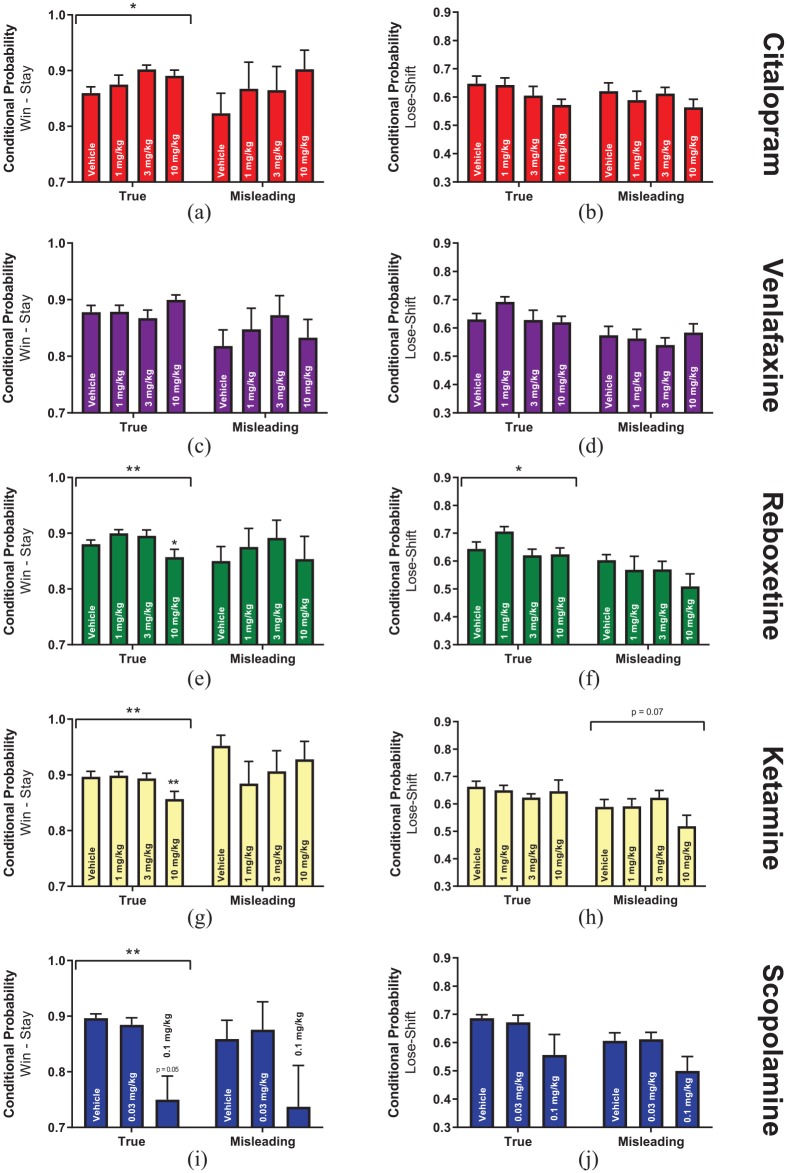
Effects of antidepressant treatment upon true versus misleading feedback sensitivity. Responses were divided as to whether they met the underlying rule of the task (true feedback) or clashed with the underlying rule (misleading feedback) the animal was required to learn at the time. (a) Citalopram: Win-stay probability. (b) Citalopram: Lose-shift probability. (c) Venlafaxine: Win-stay probability. (d) Venlafaxine: Lose-shift probability. (e) Reboxetine: Win-stay probability. (f) Reboxetine: Lose-shift probability. (g) Ketamine: Win-stay probability. (h) Ketamine: Lose-shift probability. (i) Scopolamine: Win-stay probability. (j) Scopolamine: Lose-shift probability. A bracket and star(s) over multiple bars indicates a main effect of treatment within a feedback type (e.g. misleading lose-shift) for a single drug study. This was only assessed when a significant interaction between treatment and feedback type was observed in the two-way ANOVA for both feedback types combined.

Recent evidence has suggested that pharmacological treatment can differentially effect the way animals respond to probabilistic rewards depending on whether they agree or disagree with the animals’ expectation of task feedback (Drozd et al., 2018; Rychlik et al., 2017). This has been described corresponding to either true or misleading feedback. We, therefore, re-analysed win-stay and lose-shift data to observe if acute antidepressant treatment in the PRLT differentially effects responses to the different feedback types. There was an inconsistent response to feedback type between experiments with the only difference found for the win-stay responses between true and misleading positive feedback for the venlafaxine study (two-way ANOVA, F_1,10_ = 8.00, p = 0.018, Figure 4(c)). There was a more consistent difference in lose-shift probability between true and misleading negative feedback with venlafaxine (two-way ANOVA, F_1,10_ = 32.72, p = 0.0002, Figure 4(d)), reboxetine (two-way ANOVA, F_1,10_ = 19.81, p = 0.001, Figure 4(f)) and ketamine studies (two-way ANOVA, F_1,9_ = 8.72, p = 0.016, Figure 4(h)) showing a decreased lose-shift probability following misleading feedback compared to true feedback. An interaction between ketamine treatment and feedback type was also observed for animal’s lose-shift response to true and misleading feedback (two-way ANOVA, F_3,27_ = 3.565, p = 0.027, Figure 4(h)). Further analysis revealed no effect of ketamine treatment on true lose-shift behaviour but a trend towards decreased sensitivity to NFS following misleading feedback emerged (RM-ANOVA, F_3,27_ = 2.69, p = 0.066). Across all studies, no main effects of drug treatment were observed, although trends were observed towards decreased win-stay behaviour following reboxetine (two-way ANOVA, F_3,30_ = 2.67, p = 0.066, Figure 4(e)) and scopolamine (two-way ANOVA, F_2,14_ = 3.17, p = 0.073, Figure 4(i)) treatment alongside a trend towards decreased lose-shift behaviour following citalopram treatment (two-way ANOVA, F_3,33_ = 2.62, p = 0.067, Figure 4(b)).

### Q-learn modelling of antidepressant treatment in the PRLT

**Figure 5.**
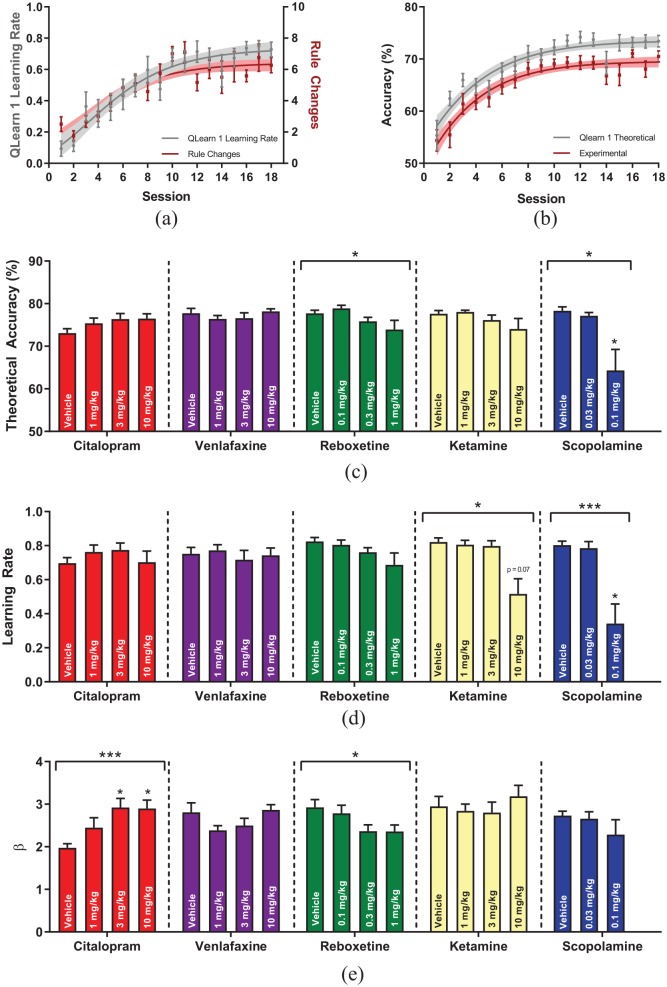
Reinforcement learning modelling of PRLT behaviour. (a) Correlation between rule changes and model-free learning rate over the first 18 sessions of training in the PRLT. (b) Correlation between absolute accuracy and accuracy compared to a model-predicted perfect strategy in the first 18 sessions of training. (c) Theoretical accuracy. (d) Model-free learning rate. (e) β, the inverse softmax temperature.

The Q-learning model was first used to analyse training data where the parameter learning rate followed the same relationship as the behavioural outcome rule changes, a measure of overall RL performance (Figure 5(a)). Theoretical accuracy, the accuracy of animals compared to a model-predicted optimal strategy, also followed a close relationship with the overall accuracy of animals in the task (Figure 5(b)), with animals always following a close but non-optimal strategy compared to ideal.

Acute dose–response study data was analysed using the Qlearn1 model due to it being the better fitting model. Both reboxetine (RM-ANOVA, F_3,30_ = 3.14, p = 0.04, Figure 5(c)) and scopolamine (RM-ANOVA, F_1.247,8.728_ = 8.77, p = 0.013) decreased theoretical accuracy, the degree to which rats’ behaviour deviated from an optimal choice strategy. Ketamine (RM-ANOVA, F_1.31, 13.10_ = 7.41, p = 0.013, Figure 5(d)) and scopolamine (RM-ANOVA, F_2,14_ = 12.36, p = 0.0008) both decreased the learning rate, the degree to which new evidence is used to make decisions as opposed to previously stored information. Softmax β, a measure of how deterministic animals’ stimulus choices are, was increased by citalopram treatment (RM-ANOVA, F_3,33_ = 7.24, p = 0.0007, Figure 5(e)). Conversely, stimulus selection was more random (lower β) when animals were treated with reboxetine (RM-ANOVA, F_3,30_ = 4.13, p = 0.014).

## Discussion

### Conventional antidepressants

Citalopram was the only conventional antidepressant tested exhibiting behavioural effects consistent with its antidepressant role. Treated animals required fewer trials to learn the first probabilistic rule, exhibited increased PFS and deterministic stimulus selection but did not show any increase in rule changes. This lack of effect upon rule changes matches escitalopram data from Drozd et al. (2018) who found no effect upon any parameter measured. However, Bari et al. (2010) observed that acute 5-mg/kg citalopram administration increased rule changes and decreased NFS, while 10-mg/kg chronic administration also increased PFS. Differences in results between the current study, Drozd et al. (2018) and Bari et al. (2010) may be related to animals’ level of performance prior to drug study experiments, with animals in this study and Drozd et al. (2018) performing roughly triple the baseline rule changes compared to Bari et al. (2010).

Reboxetine decreased RL performance and PFS. Noradrenaline has been observed to support choice variability with clonidine-treated monkeys, a manipulation reducing central nervous system (CNS) noradrenaline levels, displaying decreased choice variability in a sequential cost/benefit decision-making task (Jahn et al., 2018). Reboxetine-treated animals in the present study had a decreased β parameter value, a proxy of increased choice variability. In the PRLT too high a choice variability could impair the ability of animals to persevere at a stimulus for long enough to reverse (Delgado et al., 2011). However, in a fixed reward probability reversal learning task, rats administered with the NET-biased tricyclic antidepressant (TCA) desipramine showed increased reversal learning performance (Seu and Jentsch, 2009). Potentially, increased noradrenaline is detrimental to performance where rules are uncertain but beneficial when the reward contingencies are deterministic.

Concurrent with results from Drozd et al. (2018), acute venlafaxine treatment did not change RL or feedback sensitivity. The lack of effect observed in the present study may be because of the mixed serotonergic and noradrenergic transporter affinities of the drug whereby an RL impairment caused by enhanced noradrenergic transmission is balanced by increased serotonergic signalling improving RL ability.

In the ABT, citalopram, venlafaxine and reboxetine all positively bias the valuation of reward during new learning over multiple days (Stuart et al., 2013). However, unless they are dosed chronically, acute conventional antidepressants do not positively bias the interpretation of an ambiguous cue in the JBT (Hales et al., 2017). These results combined with the observations in the present study suggest that conventional antidepressants do not alter absolute RL, rather requiring at least overnight integration of memories to have an effect.

### Rapid-acting antidepressants

Both ketamine and scopolamine impaired RL, PFS and motivation. Both drugs have sedative effects at higher doses, a potential cause of their motivational impairments, although it has been observed that motivation and RL are dissociable within the PRLT (Roberts et al., 2019). Rychlik et al. (2017) argued that attenuating animals’ sensitivity to misleading negative feedback may be a mechanism for ketamine’s therapeutic action. We also observed an interaction between ketamine treatment and feedback type with a trend towards decreased misleading NFS. However, interpreting the significance of this finding is difficult when compared with the marked impairments ketamine had upon overall probabilistic learning and PFS. No such interaction was seen in animals treated with scopolamine.

Other studies have also suggested ketamine impairs reward processing. Administration of ketamine to both rats (10 mg/kg) and humans (0.5 mg/kg) has been found to reduce reward anticipation responses in the ventral striatum to either money or food (Francois et al., 2016). In addition, it has been observed that ketamine (32 mg/kg) impairs rats’ ability to assign a motivational value to a reward-predicting cue (Fitzpatrick and Morrow, 2017). Scopolamine’s ability to impair RL has been observed in other tasks, with an acute 0.17-mg/kg dose increasing error rate upon rule reversal in a non-probabilistic reversal learning task in mice (Pelsőczi and Lévay, 2017).

In similar low doses to those tested here, work in our laboratory has previously observed that ketamine has a robust effect on ambiguous cue interpretation and retrieval of memory biases in the ABT at 1 mg/kg (Hales et al., 2017; Stuart et al., 2015). These results combined with observations from the present study seem to suggest that while ketamine administration impairs RL, in assays of affective bias, it modifies biases during retrieval and positively biases interpretation of ambiguous cues. With regard to scopolamine, one could tentatively conclude that scopolamine appears to impair RL; however, more research is needed.

### Reinforcement learning model

When fitting behavioural data with the Q-learn model, we observed that the single learning rate Q-learning model fits best, irrespective of drug treatment. This is in contrast to previous PRLT studies in rodents where a dual learning rate model was the best fitting (Alsiö et al., 2019; Noworyta-Sokolowska et al., 2019). This also differs to human data where a dual rate model is again better fitted (Grogan et al., 2017). One possibility for the difference in best fitting model might be due to subtleties in the training for the task resulting in animals within different studies using different strategies to perform the task. The fact that theoretical accuracy was consistently higher than actual task accuracy suggests that there are factors underlying animal performance that are not accounted for in the Q-learning model tested here. Potential solutions to this could include utilising other models such as those employing choice stickiness (Alsiö et al., 2019) or fictitious updating of the non-chosen option (Noworyta-Sokolowska et al., 2019).

Citalopram increased the β parameter, implying choices were more deterministic, while reboxetine led to animals making more random choices. Citalopram’s ability to increase deterministic choice selection is interesting in the context of citalopram acutely increasing anxiety (Urban et al., 2016) and suggests that animals were better able to form and execute strategy to perform the task. Reboxetine and scopolamine both impaired theoretical accuracy, while reboxetine also decreased the β parameter; these impairments are consistent with their effects upon rule changes. Ketamine and scopolamine decreased the learning rate with this effect again mapping onto their impairments upon rule change performance. One recent study found that ketamine administration in humans performing a PRLT containing a risk-based element caused no change in the learning rate but impaired ability to follow an optimal reward strategy (similar to theoretical accuracy, Vinckier et al., 2016). Surprisingly, it has been observed that learning rate is negatively correlated with reward sensitivity and that patients with MDD or high anhedonia have decreased reward sensitivity but no change in the learning rate (Huys et al., 2013). This would suggest that for drugs to have antidepressant efficacy, they cannot increase both the learning rate and reward sensitivity. This would mean that the ketamine-mediated decrease in both the learning rate and PFS is more likely due to a general impairment of cognitive functioning as opposed to a specific effect on RL.

### Comparison with human literature

Human PRLT studies have observed that escitalopram and citalopram increase both errors made to criterion while achieving the first reversal and misleading NFS (Chamberlain et al., 2006; Skandali et al., 2018). Tryptophan supplementation, a manipulation increasing synaptic serotonin levels, has been also found to have no effect upon reversal learning errors in the PRLT (Thirkettle et al., 2019). Interestingly, the SSRI paroxetine has been found to attenuate the bias that depressed patients have towards preferentially learning from negative feedback compared to positive feedback (Moustafa et al., 2013). Atomoxetine, a noradrenaline reuptake inhibitor, has also been observed in the human PRLT to have little effect on both misleading negative feedback and errors to criterion (Chamberlain et al., 2006; a similar measure to trials to first rule change), similar to the results seen in rodents. There is a lack of human studies examining any of the other drugs tested in the present study.

Although the translatability of the PRLT is one of its key strengths, there are noticeable differences between how humans and rodents complete the task. Aside from the previously discussed differences in model fitting between species, there are also marked differences in baseline feedback sensitivity. In response to misleading negative feedback, humans rarely switch to the opposite stimulus (p(lose-switch) = 0.05, Skandali et al., 2018) compared to rats in this study (p(lose-switch) ≈ 0.65). Humans are also able to dissociate between true and misleading positive feedback (win-stay following misleading positive feedback: p(win-stay) = 0.01 and true positive feedback: p(win-stay) = 0.86), while rodents in this study were not able to do this. This implies that rodents and humans are potentially using different strategies to complete the task, meaning interpretation of the results between species might not be straightforward.

### Summary

These data suggest that within this PRLT, antidepressant treatment does not consistently modify RL or feedback sensitivity in a manner congruent with the drugs’ antidepressant efficacy or time course of effects. Citalopram’s ability to improve RL and modify PFS in the face of other drugs causing general impairments suggests that the task is potentially sensitive to manipulations of serotonergic neurotransmission. These results also add further weight that motivation and RL are dissociable within the PRLT due to the ability of citalopram to improve RL but impair motivation. Unlike tasks which measure affective biases, this PRLT did not reveal any differences between the rapid-acting and conventional antidepressants which could be related to the temporal differences in their clinical benefits. Evidence from reinforcement learning model analysis also suggested that none of the drugs improved innate reward processing parameters. All the drugs tested are effective antidepressants but, except for citalopram, either caused general impairments in RL or had no effect in this task. This suggests that modulation of RL and feedback sensitivity, as measured in this task, does not appear to be a key mechanism for their therapeutic efficacy.

## Supplemental Material

Wilkinson_PRLT_-_Supplementary_data – Supplemental material for Comparison of conventional and rapid-acting antidepressants in a rodent probabilistic reversal learning task Click here for additional data file. Supplemental material, Wilkinson_PRLT_-_Supplementary_data for Comparison of conventional and rapid-acting antidepressants in a rodent probabilistic reversal learning task by Matthew P. Wilkinson, John P. Grogan, Jack R. Mellor and Emma S. J. Robinson in Brain and Neuroscience Advances
