# Supplementary material for: Comparison of conventional and rapid-acting antidepressants in a rodent probabilistic reversal learning task
Source: Brain Neurosci Adv. 2020 Feb 23;4:2398212820907177. doi: 10.1177/2398212820907177 (PMC7085917; doi:10.1177/2398212820907177)
Supplement: Wilkinson_PRLT_-_Supplementary_data – Supplemental material for Comparison of conventional and rapid-acting antidepressants in a rodent probabilistic reversal learning task [file Wilkinson_PRLT_-_Supplementary_data.docx]

**Supplementary Material**


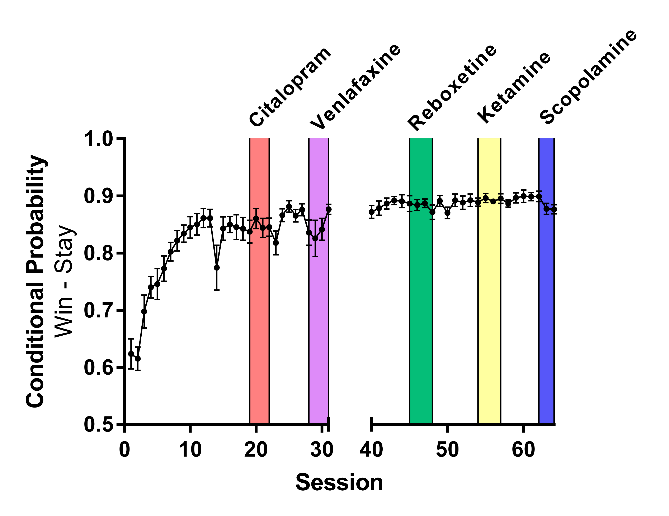

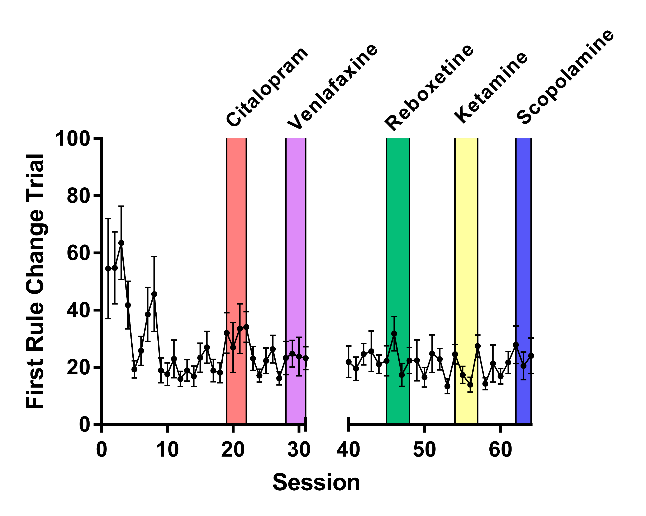

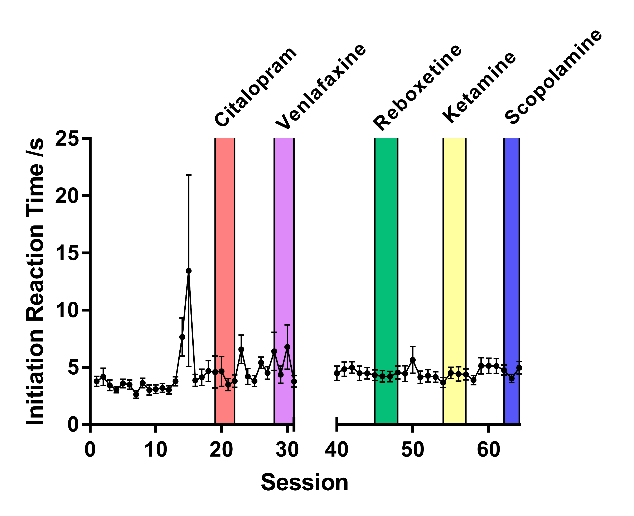

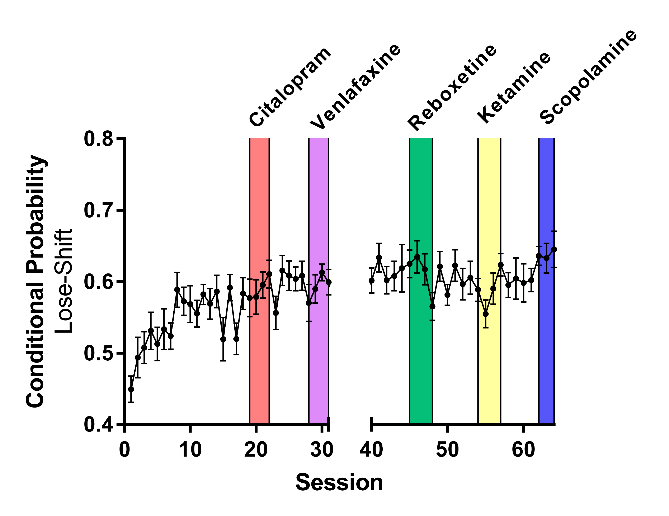

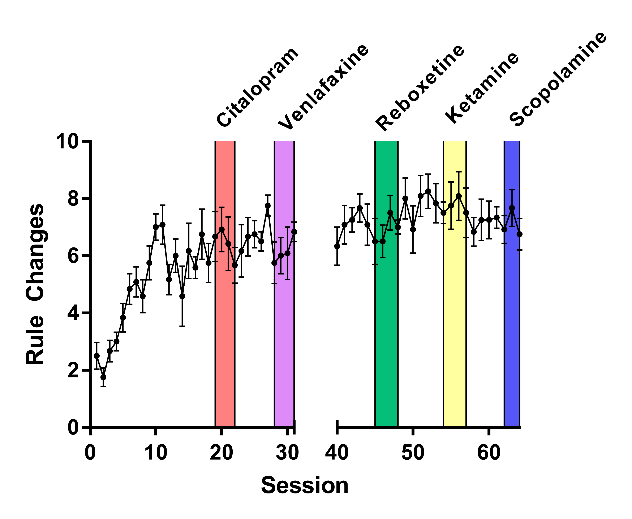


**Figure S1. Training of animals in the PRLT.** Graphs showing the training of animals from their initial first session in the probabilistic reversal learning task paradigm up to and including their participation in the final scopolamine study. **(A)** Rule changes completed within a session. (**B**) Trial at which animals first met criterion for a rule change. (**C**) Win-stay probability (**D**) Lose-shift probability. (**E**) Initiation reaction time.


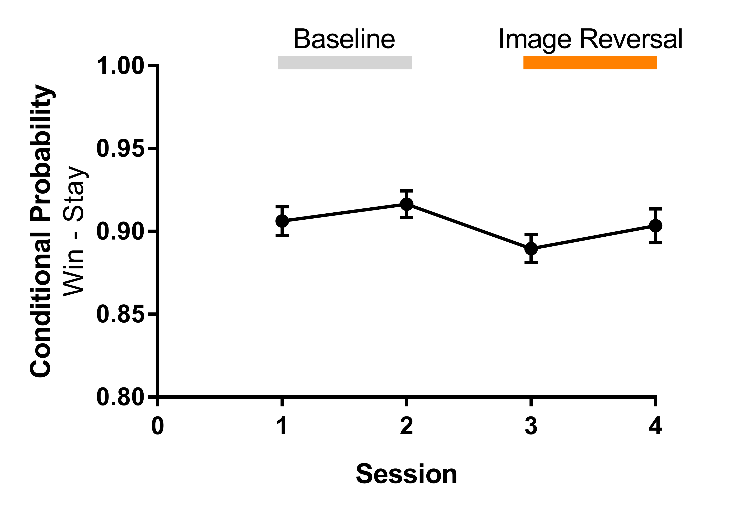

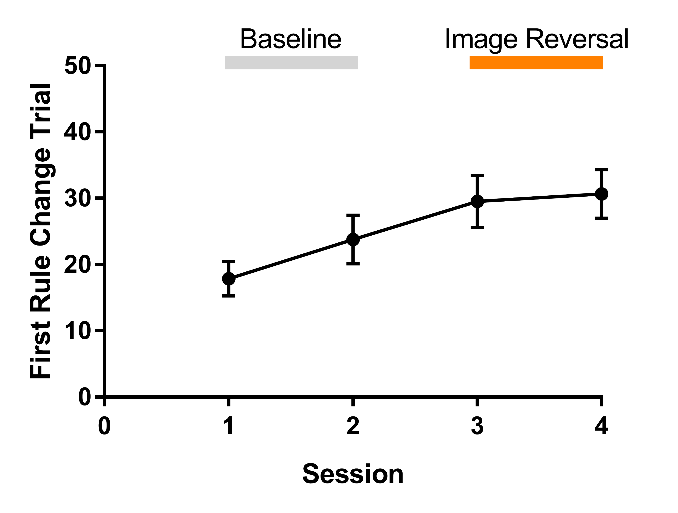

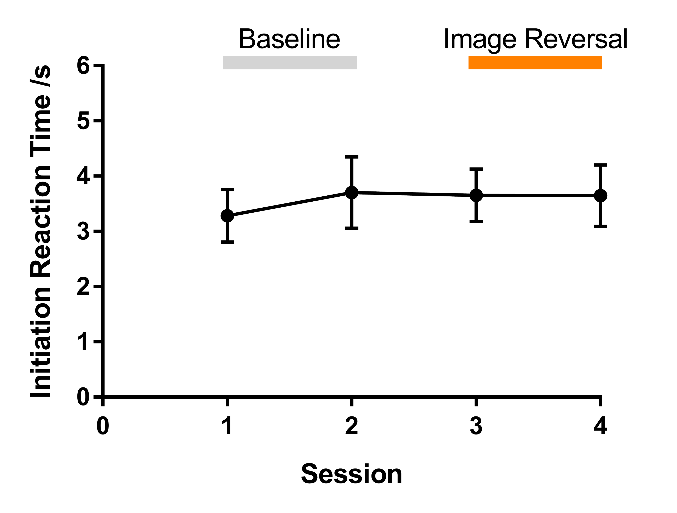

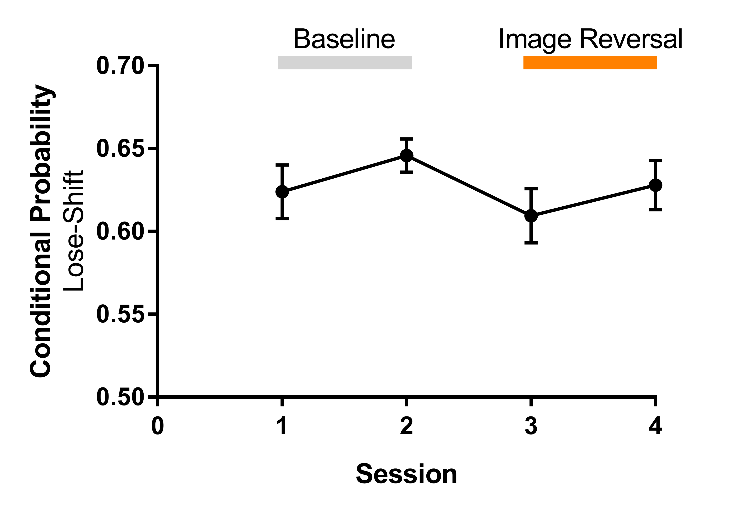

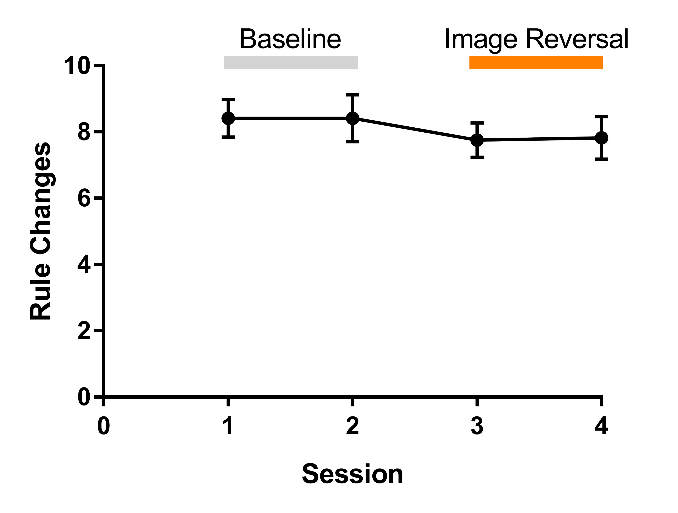


**Figure S2. Effects of reversing the starting reward location.** After two sessions of baseline recording the spatial location that initially was the rich stimulus when animals first start a session was switched to the opposite spatial location. **(A)** Rule changes completed within a session. (**B**) Trial at which animals first met criterion for a rule change. (**C**) Win-stay probability (**D**) Lose-shift probability. (**E**) Initiation reaction time. There were no statistical main effects associated with changing of the starting spatial location.


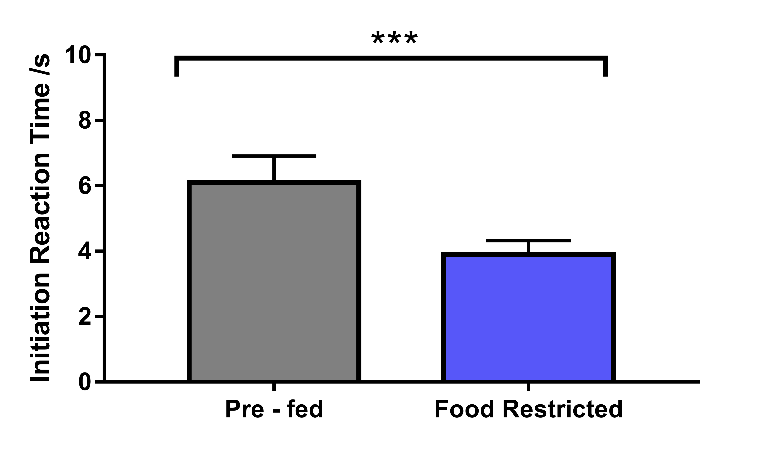

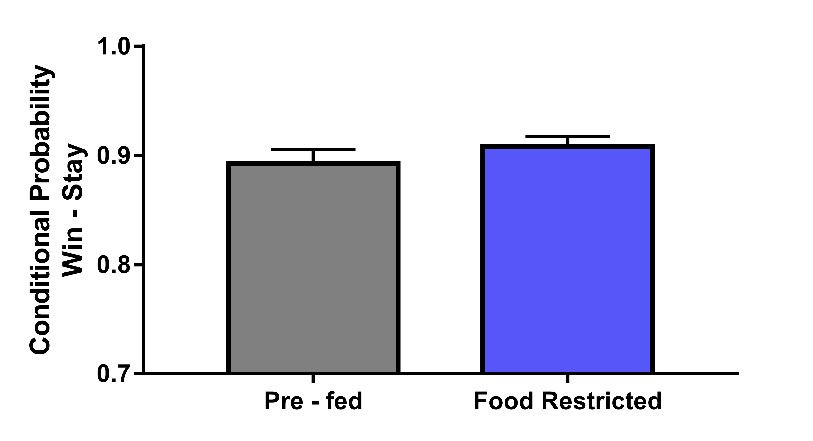

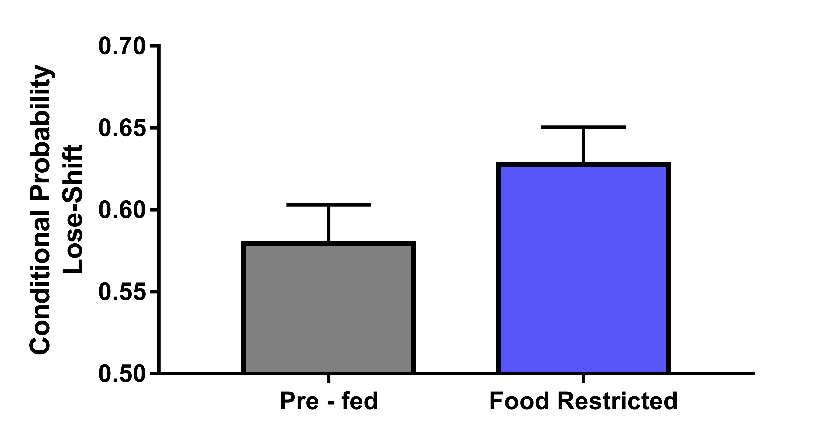

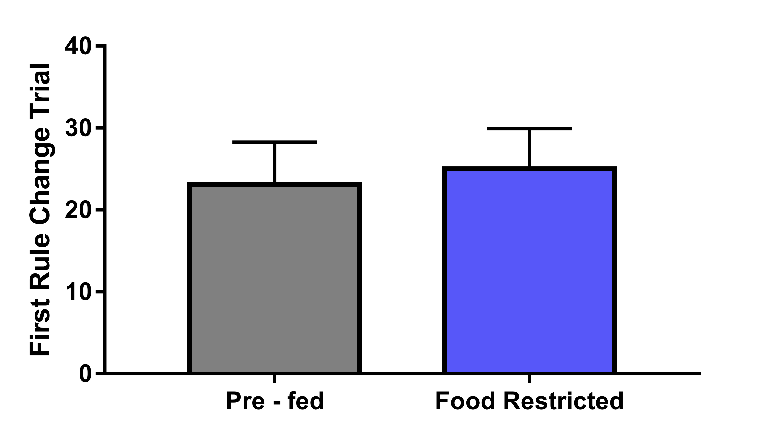

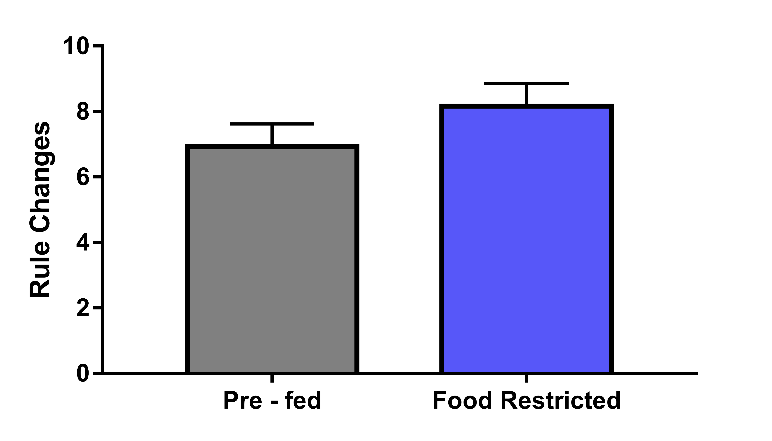


**A**

**C**

**E**

**B**

**D**

**Figure S3. Effects of pre-feeding animals before testing.** Over two sessions animals were either fed after the session (food-restricted) or before the session (pre-fed) in a counterbalanced manner. **(A)** Rule changes completed within a session. (**B**) Trial at which animals first met criterion for a rule change. (**C**) Win-stay probability (**D**) Lose-shift probability. (**E**) Initiation reaction time (Paired t-test, t_11_ = 4.6, p = 0.0008).


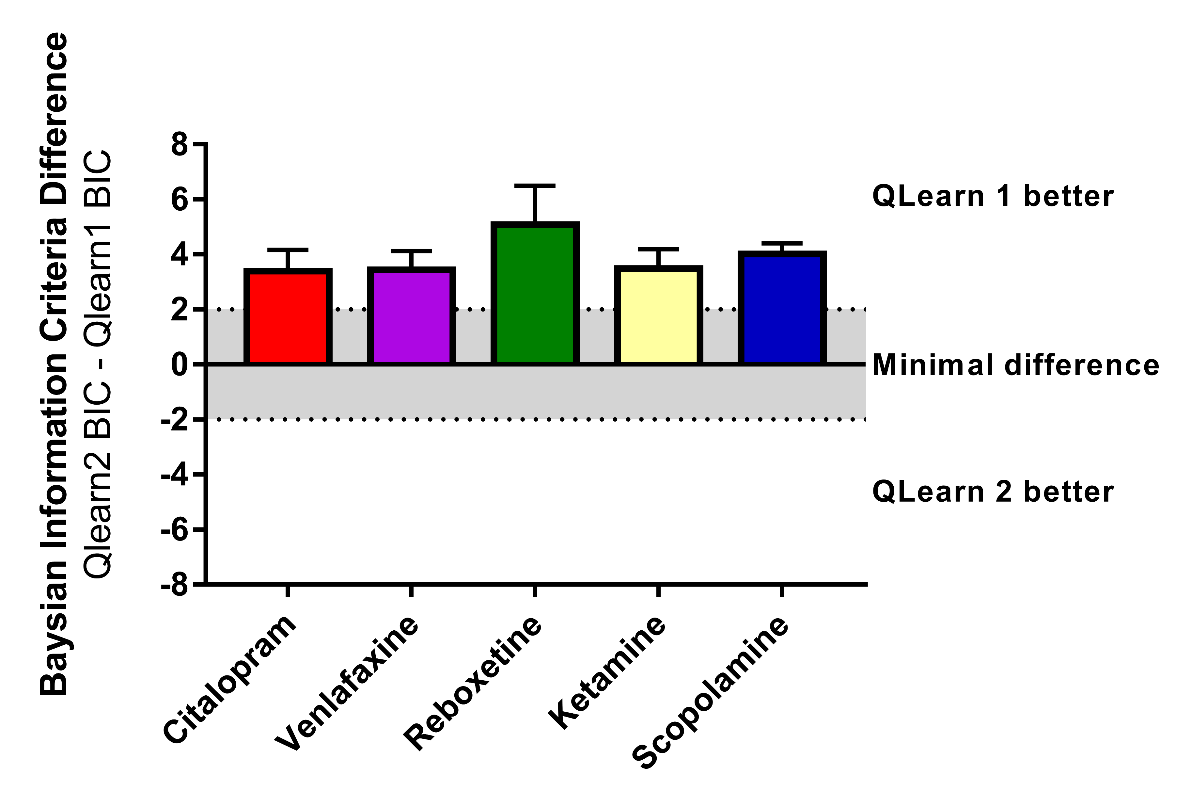


**Figure S4. Comparison of fits between Qlearn1 and Qlearn2 model.** The model fit for each drug study was assessed by fitting vehicle data with both Qlearn1 and Qlearn2 models and then using Bayesian information criteria (BIC) to choose the best fitting model. Shown is the difference between BICs between the Qlearn1 and Qlearn2 models for each drug study. A difference in BIC between models (ΔBIC) of -2 ≥ x ≤ 2 is interpreted as there being little difference in model fit. A ΔBIC ≤ -2 indicates that the Qlearn2 model fit better while a ΔBIC ≥ 2 indicates that the Qlearn1 model fit better.


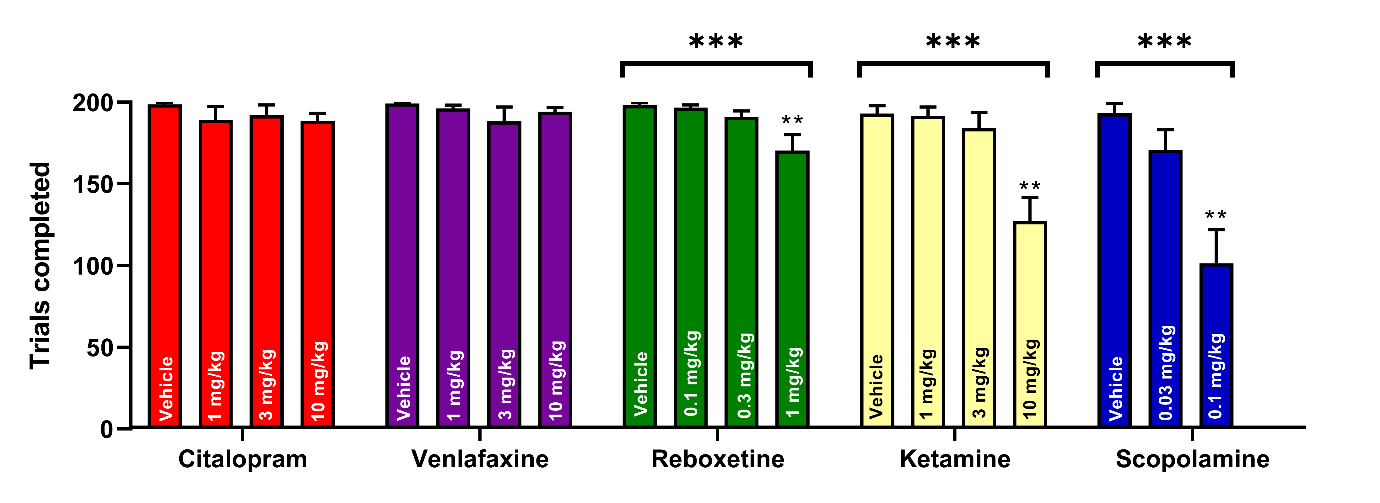


**Figure S5. Effect of antidepressant administration upon trials completed in the PRLT.** Animals performed the task until 200 trials were performed or 40m elapsed. Reboxetine, ketamine and scopolamine all reduced the number of trials completed in a session (Friedman test, reboxetine: χ2(3) = 18.0, p = 0.0004, ketamine: χ2(3) = 19.08, p = 0.0003, scopolamine: χ2(2) = 14.6, p = 0.0007)
